# Biochemical evaluation of the anticancer potential of the polyamine-based nanocarrier Nano11047

**DOI:** 10.1371/journal.pone.0175917

**Published:** 2017-04-19

**Authors:** Tracy Murray-Stewart, Elena Ferrari, Ying Xie, Fei Yu, Laurence J. Marton, David Oupicky, Robert A. Casero

**Affiliations:** 1 The Sidney Kimmel Comprehensive Cancer Center at Johns Hopkins University, Baltimore, Maryland, United States of America; 2 Center for Drug Delivery and Nanomedicine, Department of Pharmaceutical Sciences, University of Nebraska Medical Center, Omaha, Nebraska, United States of America; 3 Department of Laboratory Medicine, University of California, San Francisco, California, United States of America; Universite du Quebec a Trois-Rivieres, CANADA

## Abstract

Synthesizing polycationic polymers directly from existing drugs overcomes the drug-loading limitations often associated with pharmacologically inert nanocarriers. We recently described nanocarriers formed from a first-generation polyamine analogue, bis(ethyl)norspermine (BENSpm), that could simultaneously target polyamine metabolism while delivering therapeutic nucleic acids. In the current study, we describe the synthesis and evaluation of self-immolative nanocarriers derived from the second-generation polyamine analogue PG-11047. Polyamines are absolutely essential for proliferation and their metabolism is frequently dysregulated in cancer. Through its effects on polyamine metabolism, PG-11047 effectively inhibits tumor growth in cancer cell lines of multiple origins as well as in human tumor mouse xenografts. Promising clinical trials have been completed verifying the safety and tolerance of this rotationally restricted polyamine analogue. We therefore used PG-11047 as the basis for Nano11047, a biodegradable, prodrug nanocarrier capable of targeting polyamine metabolism. Following exposure of lung cancer cell lines to Nano11047, uptake and intracellular degradation into the parent compound PG-11047 was observed. The release of PG-11047 highly induced the polyamine catabolic enzyme activities of spermidine/spermine *N*^1^-acetyltransferase (SSAT) and spermine oxidase (SMOX). By contrast, the activity of ornithine decarboxylase (ODC), a rate-limiting enzyme in polyamine biosynthesis and a putative oncogene, was decreased. Consequently, intracellular levels of the natural polyamines were depleted concurrent with tumor cell growth inhibition. This availability of Nano11047 as a novel drug form and potential nucleic acid delivery vector will potentially benefit and encourage future clinical studies.

## Introduction

**Fig 1:**
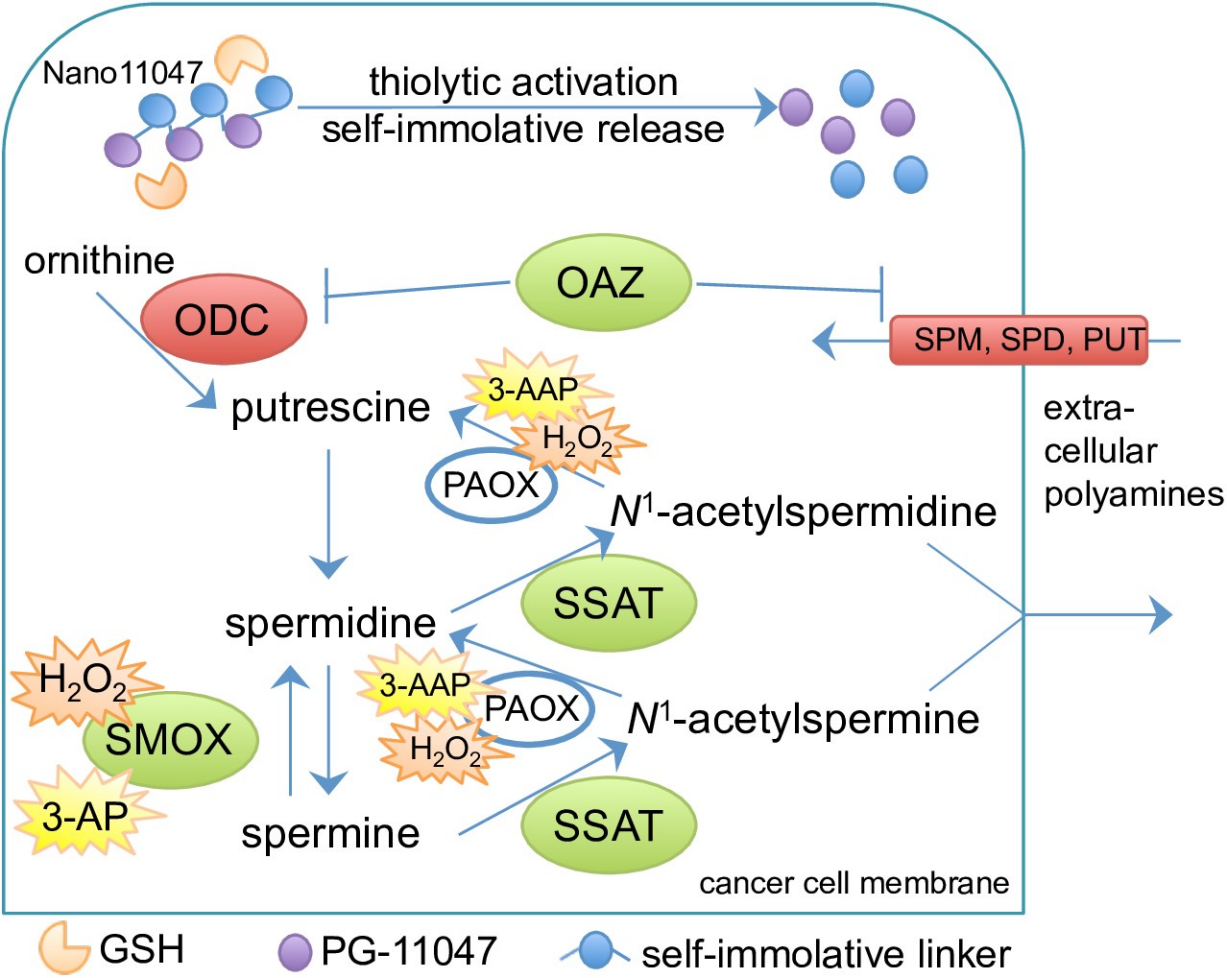
Illustration depicting the mechanism of action for polyamine nanocarriers and their effects on polyamine metabolism. Following endocytosis, Nano11047 undergoes thiolytic reduction by glutathione (GSH), followed by disassembly and release of PG-11047. Effects of intracellular PG-11047 accumulation on polyamine metabolism are indicated in green for upregulation or red for downregulation, resulting in an overall decrease in the natural polyamine pools required to sustain cancer cell proliferation. Abbreviations used in the figure are as follows: ODC, ornithine decarboxylase; OAZ, ODC antizyme; 3-AAP, 3-acetaminopropanal; 3-AP, 3-aminopropanal; PAOX, polyamine oxidase; SSAT, spermidine/spermine *N*^1^-acetyltransferase; SMOX, spermine oxidase.

The naturally occurring eukaryotic polyamines (spermine, spermidine, and putrescine) are essential for cellular proliferation, differentiation, and survival [1], and fluctuations in their intracellular levels can influence important cellular processes such as nucleosome formation, DNA replication, gene transcription, protein synthesis, membrane stability, ion channel regulation, and free radical scavenging [2–5]. The intracellular concentrations of the polyamines are therefore tightly regulated by the enzymes of the polyamine metabolic pathway as well as through regulated uptake from the extracellular environment. Importantly, polyamines are observed at elevated intracellular concentrations in proliferating cells, particularly cancer cells, due to dysregulation of their metabolism. As these elevated polyamine levels are required by tumor cells to maintain proliferation, polyamine analogues have been developed to exploit polyamine-sensitive feedback mechanisms as potential therapies for cancer and other hyperproliferative conditions [6–8]. As versions of the natural polyamines that can function in the regulation of polyamine metabolism, but not in the growth supporting functions, the symmetrically substituted bis(ethyl) class of polyamine analogues utilize the polyamine transport machinery to gain entry into cells, where they commonly upregulate the polyamine catabolic enzymes spermidine/spermine *N*^1^-acetyltransferase (SSAT) and/or spermine oxidase (SMOX), while down-regulating biosynthesis and uptake, thereby depleting the cells of the natural polyamines and causing growth arrest (**Fig 1**). Additionally, in cells that respond to analogue treatment with an induction of SMOX, generation of the reactive oxygen species hydrogen peroxide (H_2_O_2_) can result in apoptotic cell death [7–9].

Polymers have been used extensively as nanocarriers for delivery of various small molecule and macromolecule drugs. The use of polymeric nanocarriers allows favorable modulation of pharmacokinetics and tissue distribution of drugs, which often leads to reduced toxicity and improved efficacy. The ability to achieve spatiotemporal control of the drug release is another advantageous feature of polymeric nanocarriers [10, 11]. Nanocarriers can typically encapsulate multiple drug molecules, which makes them ideally suited for simultaneous delivery of drug combinations [12]. Typical nanocarriers are pharmacologically inert systems intended for conjugation or encapsulation of drugs. This traditional approach often suffers from low drug loading, which led to the development of alternative strategies utilizing polymers synthesized directly from existing drugs [13–15]. We have recently applied this concept to develop biodegradable polycations as prodrugs that release the polyamine analogue BENSpm to target polyamine metabolism and codeliver therapeutic miRNA in cancer [16]. The interaction of polyamines with nucleic acids can spontaneously induce condensation into nanosized particles, facilitating preparation and transport into the cell. Thomas and colleagues recently provided a comprehensive review of these DNA–polyamine interactions as well as polyamine-associated gene delivery vehicles [17].

**Fig 2:**
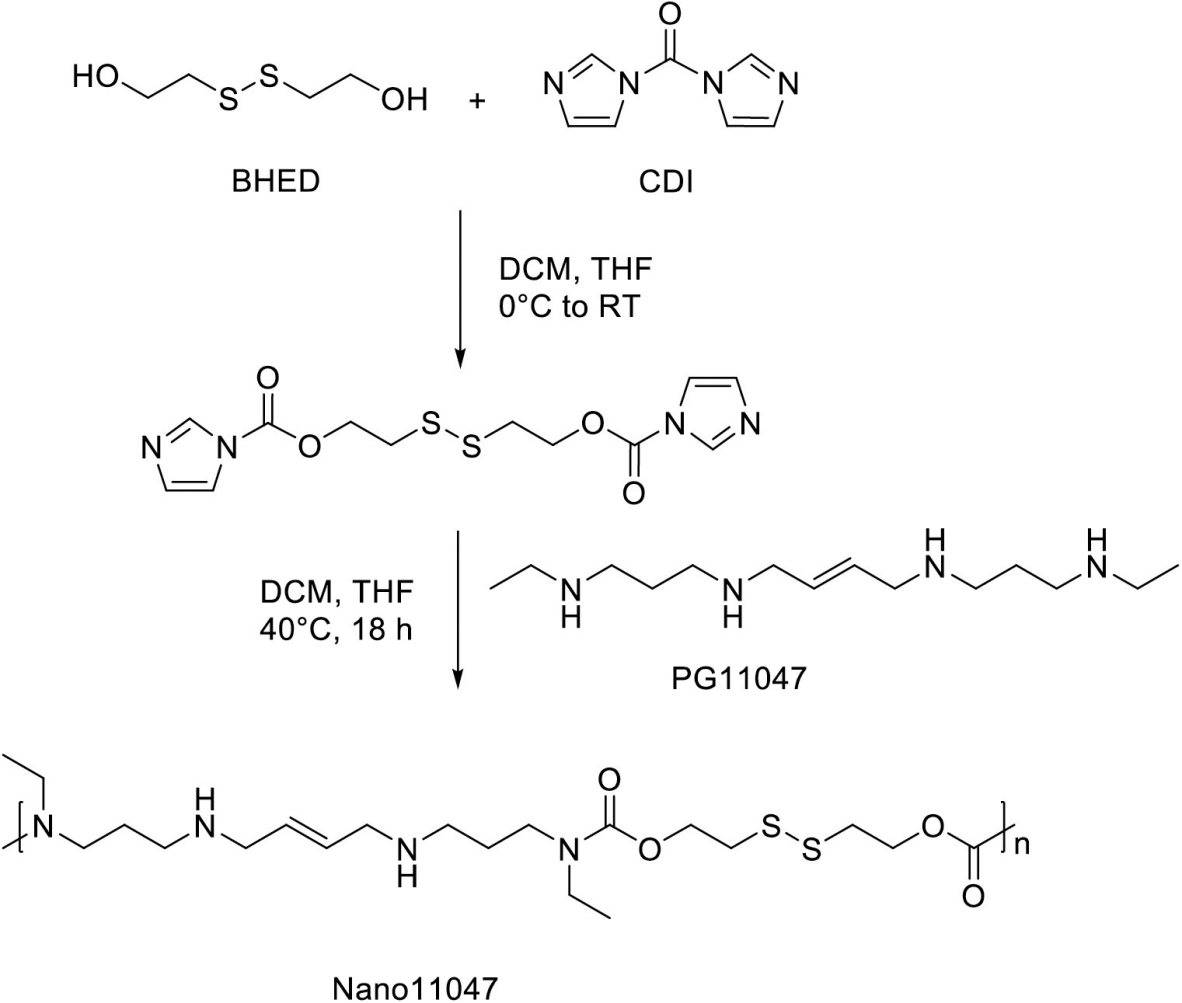
Synthesis of Nano11047.

In the current study, we investigate the antitumor potential of a biodegradable, polyamine analogue-derived nanocarrier, Nano11047, to target the polyamine metabolic pathway in human non-small cell lung carcinoma (NSCLC) cell lines. The parent compound of Nano11047, PG-11047, or *N*^1^,*N*^12^-bis(ethyl)-*cis*-6,7-dehydrospermine tetrahydrochloride, is a conformationally restricted derivative of the first-generation polyamine mimetic *N*^1^,*N*^12^-bis(ethyl)spermine (BESpm)[18]. By incorporating a *cis* double bond between its central carbons (**Fig 2**), the spatial rigidity of PG-11047 is increased with the goal of enhancing the selective binding of polyamine targets, including nucleic acids [18]. Capable of dramatically up-regulating polyamine catabolism, down-regulating polyamine biosynthesis and uptake, depleting natural polyamines, and producing reactive oxygen species (**Fig 1**), PG-11047 treatment causes significant growth inhibition in various human cancer cell lines, including those representing lung, prostate, breast, and colon cancers, both *in vitro* and in human tumor xenograft mouse models [18–25]. Although certain tumor types, specifically small cell lung carcinomas (SCLC), tend to be resistant to the antiproliferative effects of PG-11047, recent work has demonstrated that adding a histone deacetylase inhibitor to the analogue treatment can sensitize these cells via a miRNA-mediated synergistic induction of SSAT activity [26]. Most importantly, PG-11047 has been safely administered and well tolerated in clinical trials as a single agent in patients with relapsed or refractory lymphoma (Clinical Trial #NCT00293488) and those with advanced refractory solid tumors (Clinical Trial #NCT00705653), where the MTD was 610 mg given once weekly. PG-11047 was also well tolerated in combination with common chemotherapeutic agents, including cisplatin, erlotinib, 5-fluorouracil, and bevacizumab in patients with advanced solid tumors or lymphoma (Clinical Trial #NCT00705874). This is a significant improvement over the clinical utility of BENSpm, which was limited due to dose-limiting toxicities [27]. Furthermore, recent gene expression studies of breast and colon cancer cells following PG-11047 treatment have revealed important information with regard to molecular signatures of responsive genes and indicators of which patients might potentially respond to treatment [22, 23], and work by Cirenajwis *et al*. has suggested that PG-11047 targets a putative breast cancer stem cell population, altering levels of several proteins involved in tumor progression and malignancy [25].

Our results indicate the feasibility of Nano11047 as a nanocarrier of PG-11047 capable of targeting the polyamine metabolic pathway for cancer cell growth inhibition. We verify Nano11047 uptake and disassembly with the intracellular release and accumulation of PG-11047. This accumulation results in decreased levels of ornithine decarboxylase (ODC), a rate-limiting enzyme of polyamine biosynthesis and putative oncogene, as well as increases in the polyamine catabolic enzymes SSAT and SMOX, resulting in depletion of the natural polyamine pools and growth inhibition/cytotoxicity in lung cancer cell lines. As the antitumor targets of PG-11047 are multiple, the availability of Nano11047 as a novel drug form and delivery method will potentially benefit and encourage future clinical studies of novel combination therapies.

## Results and discussion

### Synthesis and characterization of Nano11047

**Fig 3:**
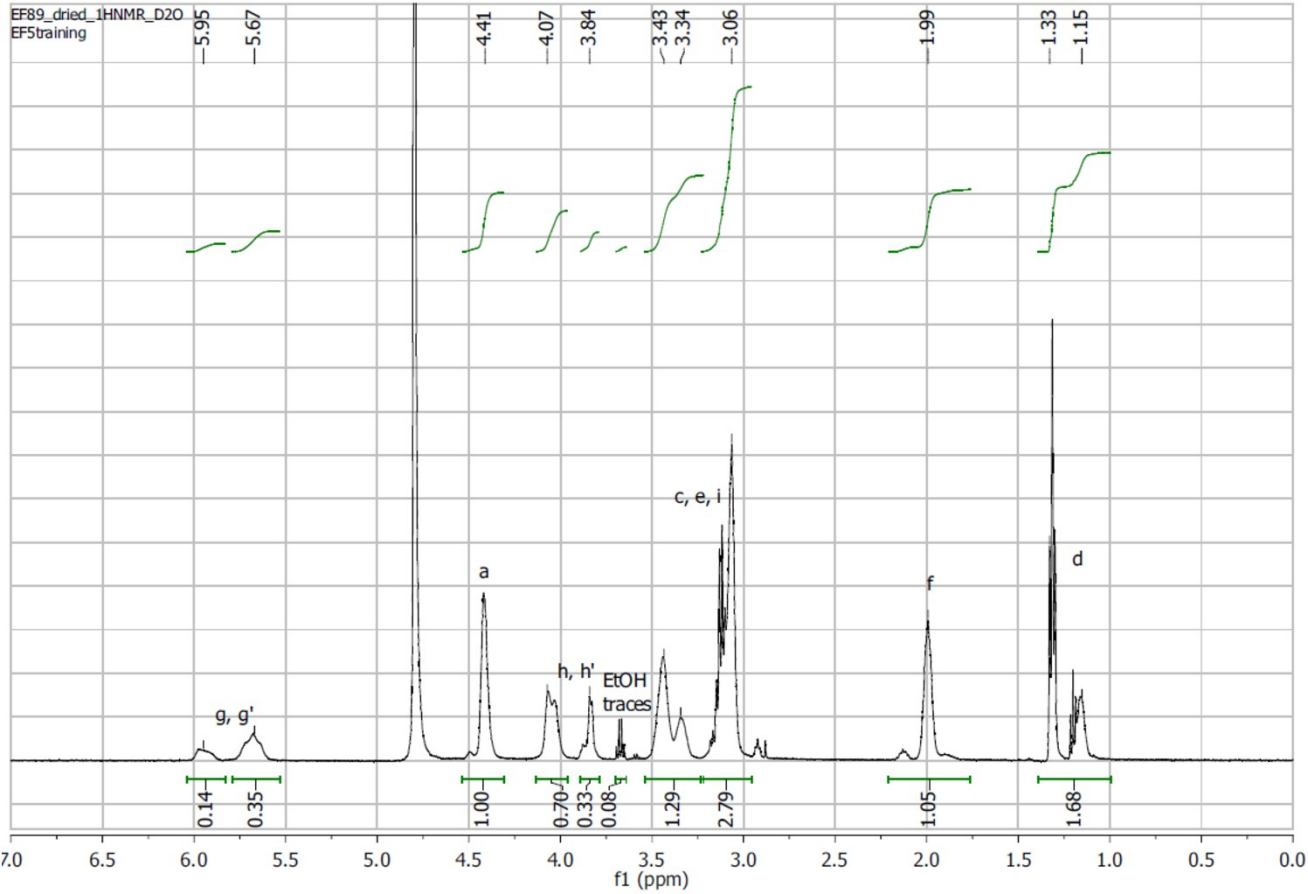
^1^H-NMR of Nano11047 in D_2_O.

We previously reported the development of self-immolative nanocarriers based on a polyamine analogue, (*N*^1^,*N*^11^)-bis(ethyl)norspermine (BENSpm) [28–30]. Due to the polycationic nature of the polyamine analogues, they readily complex with positively charged macromolecules, including RNA and DNA. Hence, BENSpm-based nanocarriers have been used successfully as delivery vectors for therapeutic genes and microRNA, while simultaneously targeting polyamine metabolism [28, 29]. Here, we further developed the concept of nanocarriers that target polyamine metabolism to the next-generation polyamine analogue PG-11047. To synthesize Nano11047, we utilized our previous approach based on a self-immolative bis(2-hydroxyethyl)disulfide (BHED) disulfide linker, which can be readily cleaved in the highly reducing intracellular environment of cancer cells. We first activated the hydroxyls of BHED with carbonyldiimidazole (CDI) and directly used the product in a subsequent reaction with free-base PG-11047 to provide Nano11047 (**Fig 2**). Nano11047 was purified by dialysis and characterized by ^1^H-NMR and size exclusion chromatography. Like BENSpm, PG-11047 consists of 4 secondary amines that can participate in the reaction. As a result, Nano11047 is obtained as a branched polymer, which was confirmed from the NMR spectrum (**Fig 3**). The molecular weight of Nano11047 was 7.2 kDa, with a PDI of 1.8. The molecular weight achievable with the conformationally restricted PG-11047 was nearly twice as high as the molecular weight obtained previously with BENSpm. The observed PDI is consistent with the polyaddition mechanism of the reaction. Further details of the NMR analysis are available in Supporting Information (S1 Fig).

### Nano11047 degradation and release of PG-11047

**Fig 4:**
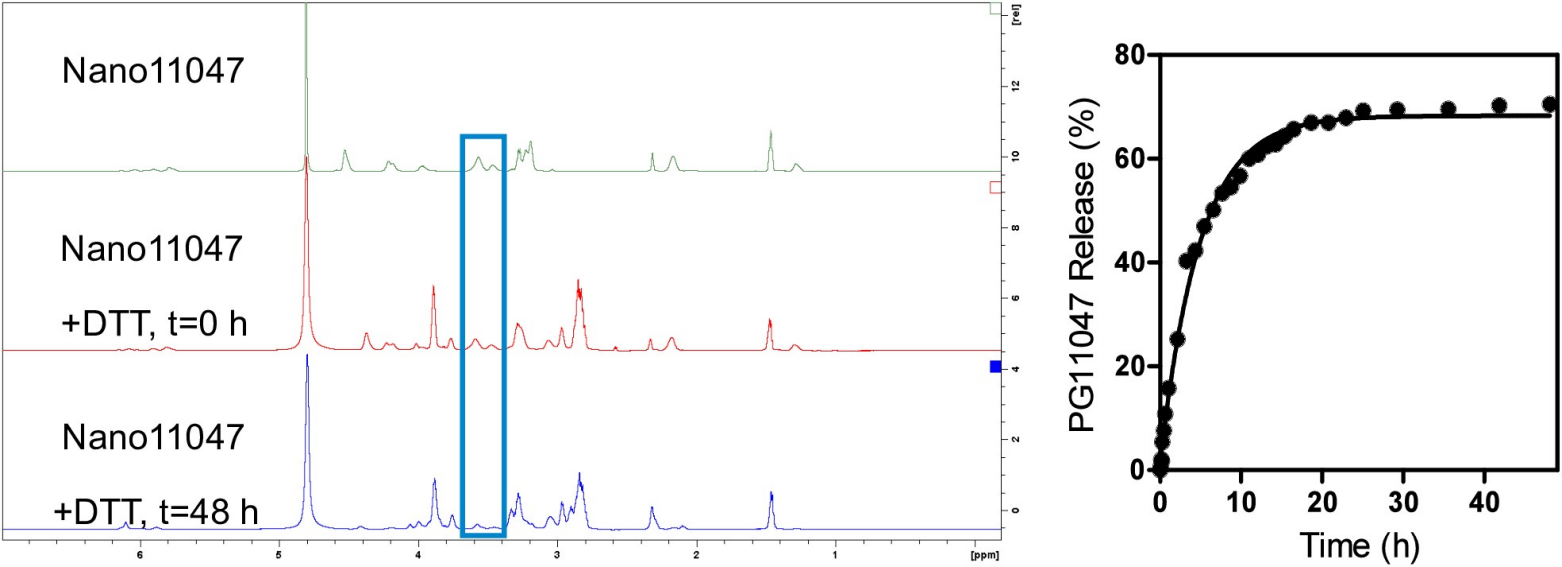
Degradation of Nano11047. Degradation of Nano11047 and PG-11047 release kinetics in 0.1 M phosphate-buffered D_2_O/acetone-*d6* (3/2, v/v, 0.9 mL, pH7.4) with 100 mM DTT at 25°C.

Intracellular cleavage of the disulfide bond in Nano11047 occurs through thiol-disulfide exchange with free thiols, predominantly reduced glutathione (GSH) [31]. This cleavage generates a free thiol, resulting in a self-immolative moiety in which the free thiol triggers an intramolecular attack on the carbamate carbonyl to release the parent PG-11047. We used DTT as a reducing agent to investigate the degradation kinetics of Nano11047. Degradation was calculated as the percentage of integral intensity decrease of the methylene peaks in the ^1^H-NMR spectra of the Nano11047 relative to the integral intensity at time 0. The results were plotted as the percent release vs. time (**Fig 4**). Analysis of the data showed that the degradation followed simple first-order kinetics with a kinetic rate constant of 3.4 × 10^−3^ min^-1^ and a half-life of 203 min.

### Effects of Nano11047 treatment on cell proliferation

**Fig 5:**
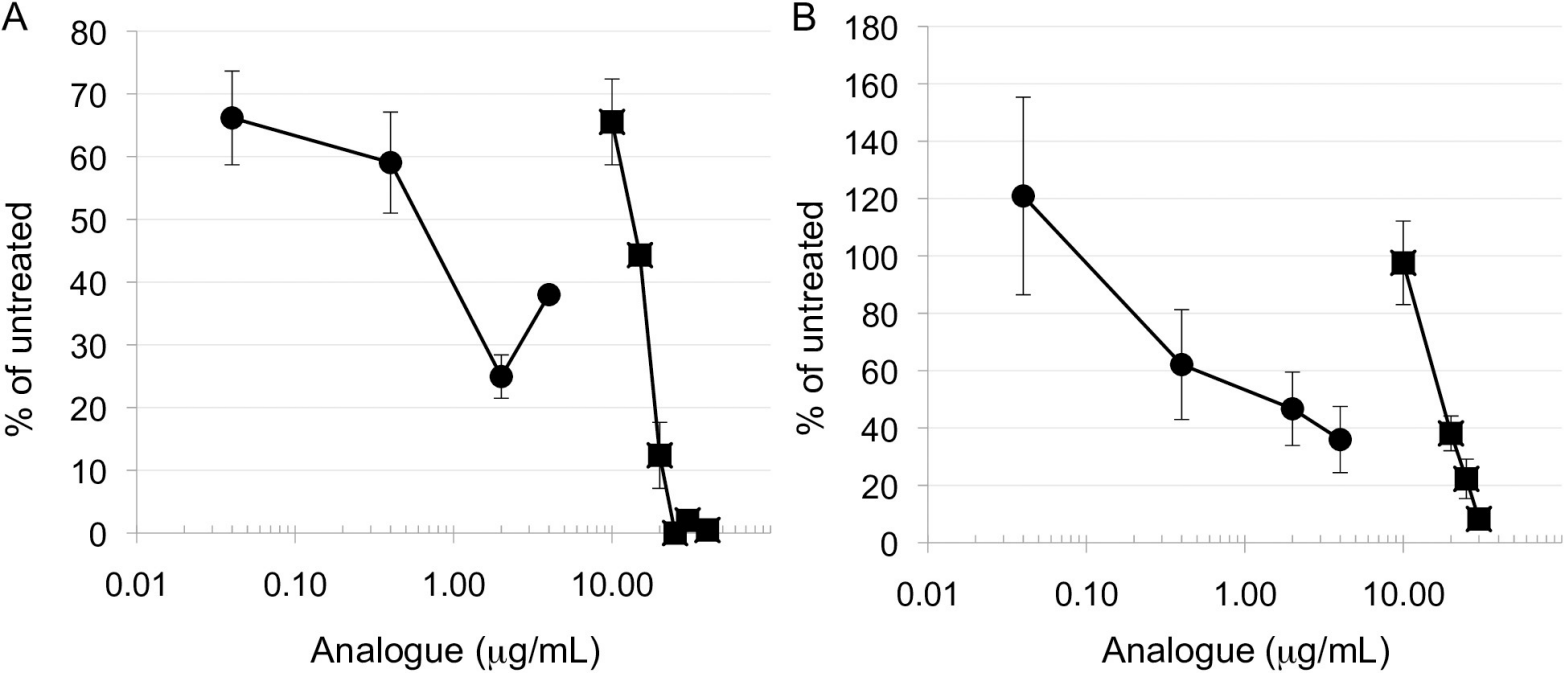
Cell viability in response to Nano11047. H157 (**A**) or A549 (**B**) NSCLC cells were treated for 96 hours with increasing concentrations of Nano11047 (■) or PG-11047 (●). Cell number was determined by hemacytometer following trypan blue exclusion. Data points represent the means with error bars indicating SEM (n = 2 experiments, each performed in duplicate).

We previously demonstrated that the bis(ethyl)polyamine analogues, including PG-11047 and BENSpm, have strong antiproliferative effects on lung cancer cell lines of the NSCLC phenotype [20, 32]. To determine if Nano11047 possessed this same potential, NCI-H157 and A549 NSCLC cells were treated with increasing doses of the nanocarrier, in parallel with PG-11047, for 96 hours. Cell counts following trypan blue exclusion revealed inhibition of cell growth in both cell lines in a concentration-dependent manner, with H157 cells displaying slightly greater sensitivity than A549 cells (**Fig 5A** vs. **5B**). It should be noted that the concentrations of Nano11047 and PG-11047 presented in Fig 5 should not be directly compared, as the concentration of Nano11047 used for treatment refers to the nanocarrier mass prior to biodegradation. For subsequent studies, concentrations of the different analogues with similar growth-inhibiting effects were used.

### Induction of spermine/spermidine *N*^1^-acetyltransferase activity by Nano11047

**Fig 6:**
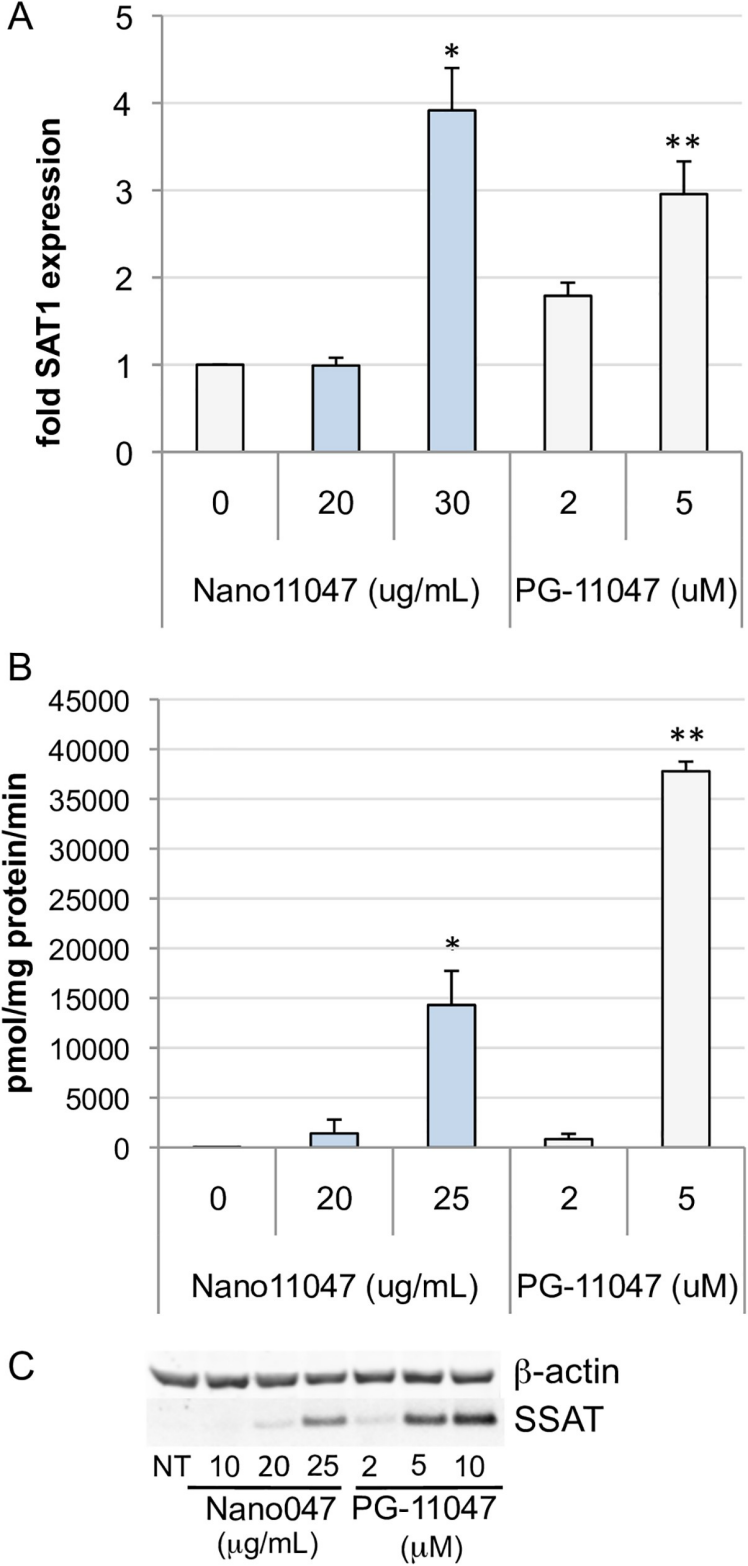
Nano11047 induces spermidine/spermine *N*^1^-acetyltransferase (SSAT) activity in H157 cells. For qRT-PCR (**A**), cells were treated 48 hours as indicated. For SSAT enzyme activity determination (**B**) and Western blot analysis (**C**), incubation time was increased to 72 h. Column heights indicate the means with error bars indicating SEM. *p < 0.05; **p < 0.005 determined by Student’s t-test, relative to untreated cells (n ≥ 2 experiments, each with triplicate determinations).

The original rationale for the use of structural polyamine analogues in cancer therapy was based on the self-regulatory nature of polyamine metabolism [33, 34], and PG-11047 has exemplified this ability in tumor cell lines of multiple origins [7, 20, 35]. Since the antiproliferative effects of the bis(ethyl) polyamines have been associated with a phenotype-specific induction of polyamine catabolism in NSCLC cells [32, 36], we examined the effects of Nano11047 exposure on the polyamine catabolic enzyme SSAT in H157 cells. Similar to results previously observed with exposure to the parent compound and others [20], these cells responded to Nano11047 with a super-induction of SSAT (**Fig 6**). SSAT protein is post-transcriptionally regulated by this class of polyamine analogues [37, 38], and Nano11047 treatment produced a dramatic increase in SSAT activity and protein (**Fig 6B and 6C**), in spite of a more modest induction of SSAT mRNA (**Fig 6A**). It should be noted that due to the cytotoxicity accompanying Nano11047 treatment, the maximal concentration was reduced when treating for SSAT activity, with a compensatory increase in incubation time.

This induction of catabolism was accompanied by a corresponding depletion in intracellular concentrations of the natural polyamines putrescine, spermine and spermidine, with accumulation of the analogue (**Table 1**). The HPLC analyses (**Table 1)** also revealed that Nano11047 was effectively reduced to PG-11047 once internalized in the cells, and it accumulated to the same extent as that in cells treated directly with a comparable dose of PG-11047. Preliminary studies using 24-h treatments of Nano11047 had no effect on the polyamine catabolic enzymes at the mRNA level, unlike PG-11047, suggesting a delay in bioavailability due to the need for Nano11047 to translocate to the cytoplasm and be degraded, which could also account for the difference in SSAT induction between the parent compound and Nano11047.

**Table 1:** Intracellular polyamine levels in H157 after a 72-h treatment.

Analogue	Concentration	*N*^1^Ac-SPD	PUT	SPD	SPM	PG-11047
none	0	1.00 (1.73)	2.48 (0.23)	2.88 (0.33)	5.05 (0.75)	ND
Nano11047	20 μg/mL	15.70 (6.39)	2.23 (0.88)	0.78 (0.21)	2.28 (0.28)	10.35 (2.38)
Nano11047	25 μg/mL	ND	0.83 (0.05)	ND	0.42 (0.11)	17.61 (2.47)
PG-11047	2 μM	8.26 (0.90)	2.35 (0.63)	0.91 (0.20)	2.78 (0.24)	5.02 (0.95)
PG-11047	5 μM	ND	ND	ND	ND	17.35 (4.39)

Polyamine concentrations are presented as nmol polyamine/mg protein. ND = none detected. Values represent means with (SEM); n = 3.

A549 cells also respond to PG-11047 treatment with a large induction of SSAT [20], and due to their slightly greater resistance to the antiproliferative effects of Nano11047, relative to H157 cells, we were able to conduct a more comprehensive analysis of the changes in intracellular polyamine concentrations with prolonged treatment. **Table 2** presents these changes following 96 hours of treatment with increasing doses of Nano11047 or PG-11047, demonstrating that Nano11047 is capable of depleting cells of the higher polyamines, spermine and spermidine. These changes, along with the accumulation of *N*^1^-acetylated spermidine at lower treatment concentrations, are indicative of the induction of SSAT. It should be noted that at higher concentrations the resultant toxicity results in less acetylated spermidine.

**Table 2:** Intracellular polyamine levels in A549 after 96-h treatment.

Analogue	Concentration	N^1^Ac-SPD	PUT	SPD	SPM	PG-11047
none	0	ND	1.25 (0.23)	17.54 (0.34)	6.54 (0.53)	ND
Nano11047	10 μg/mL	5.79 (1.54)	3.62 (0.54)	3.99 (0.16)	3.34 (0.32)	32.35 (1.60)
Nano11047	15 μg/mL	7.57 (0.10)	5.92 (0.14)	0.91 (0.03)	1.07 (0.04)	49.11 (0.24)
Nano11047	20 μg/mL	0.41 (0.09)	1.32 (0.28)	ND	ND	75.67 (7.28)
Nano11047	25 μg/mL	0.58 (0.20)	1.21 (0.20)	ND	ND	65.83 (5.58)
PG-11047	0.1 μM	ND	1.26 (0.22)	17.12 (0.80)	5.61 (0.23)	1.20 (0.11)
PG-11047	1 μM	1.32 (0.42)	2.25 (0.40)	8.11 (0.21)	4.69 (0.43)	16.20 (1.40)
PG-11047	5 μM	ND	ND	ND	ND	70.80 (3.44)
PG-11047	10 μM	ND	ND	ND	ND	72.11 (3.62)

Polyamine and analogue concentrations are presented as nmol polyamine/mg protein. ND = none detected. Values represent means (SEM); n = 4.

### Induction of spermine oxidation and H_2_O_2_ generation by Nano11047

**Fig 7:**
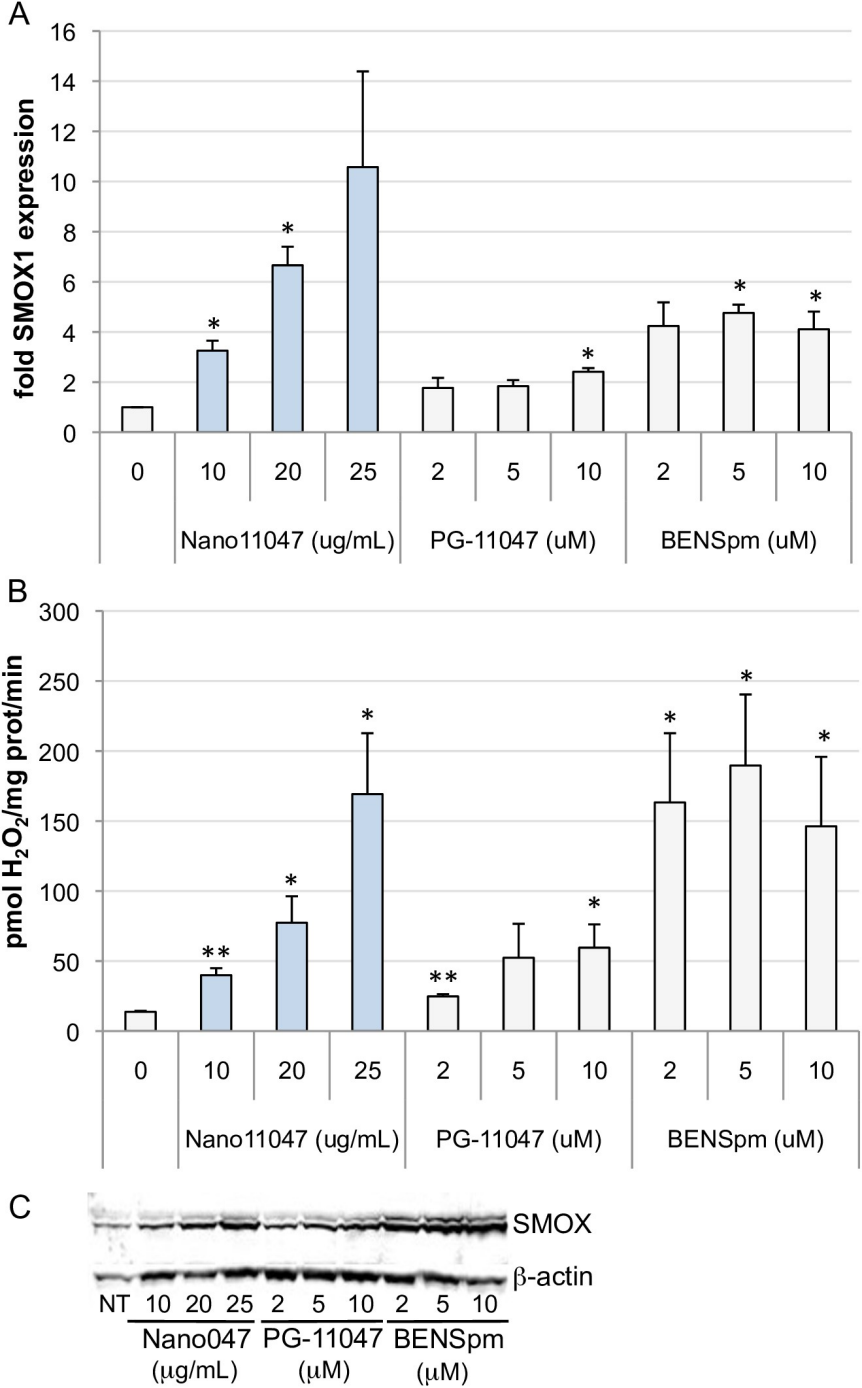
Nano11047 induces spermine oxidase (SMOX) activity. A549 cells were treated with increasing concentrations of the analogues for 48 h. SMOX mRNA expression was determined by qRT-PCR (**A**); SMOX activity (**B**) was determined based on spermine-specific production of H_2_O_2_. A representative Western blot (**C**) indicates induction of SMOX isoforms. Column heights indicate the means with error bars indicating SEM. *p < 0.05; **p < 0.005 determined by Student’s t-test, relative to untreated cells (n ≥ 2 experiments, each with triplicate determinations).

In addition to acetylation by SSAT, spermine can undergo direct oxidation by SMOX to form spermidine, H_2_O_2_, and 3-aminopropanal (3-AP). The bis(ethyl)polyamine analogues, including PG-11047, have also been shown to induce SMOX in a cell-type specific manner [20, 39]. As A549 cells have historically shown the greatest induction of SMOX, we investigated the potential for induction in these cells by Nano11047. As shown in **Fig 7**, Nano11047 induced SMOX mRNA, protein, and activity, as measured by the production of H_2_O_2_, to an extent greater than PG-11047, and similar to that of BENSpm, the most potent of the bis(ethyl)polyamine analogues in terms of polyamine catabolism induction. As SMOX-generated H_2_O_2_ is a reactive oxygen species with the potential to cause DNA damage and induce apoptosis, and the depletion of spermine, which can act as a radical scavenger [5, 40] (**Table 2**), results in a decreased antioxidant pool, this induction of SMOX presents an additional mechanism through which Nano11047 has therapeutic potential.

### Inhibition of polyamine biosynthesis

**Fig 8:**
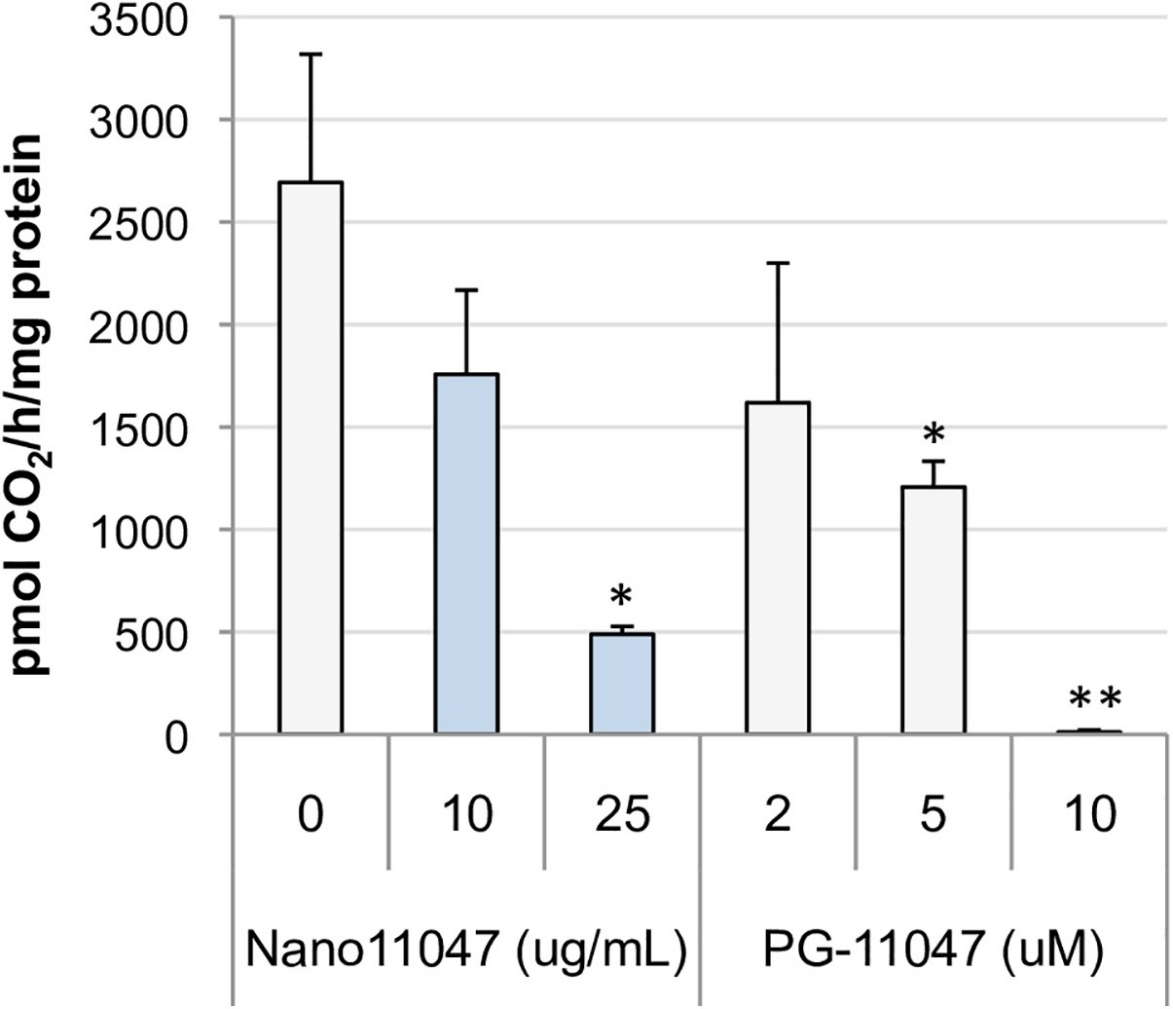
Nano11047 down-regulates ornithine decarboxylase (ODC) activity. H157 cells were treated as indicated for 72 h, and ODC enzyme activity was determined through the decarboxylation of radiolabelled ornithine. Column heights indicate the means with error bars indicating SEM. *p < 0.05; **p < 0.005 determined by Student’s t-test, relative to untreated cells (n = 2 experiments with triplicate determinations).

Ornithine decarboxylase (ODC) is the first rate-limiting polyamine biosynthetic enzyme. Due to its ability to promote proliferation and its position as a downstream effector of Myc, ODC is also considered a putative oncogene [41]. As part of their antitumor capabilities, the bis(ethyl)polyamine analogues also commonly down-regulate ODC via their ability to induce antizyme, a negative regulator of ODC [20, 42]. In the current study, Nano11047 effectively down-regulated ODC activity in H157 cells to less than 20% of the level of untreated cells, as indicated in **Fig 8**.

## Conclusions

In conclusion, we have shown in solid tumor, lung cancer cell lines, that the nanocarrier version of PG-11047, Nano11047, can stimulate polyamine catabolism, decrease polyamine biosynthesis, produce reactive oxygen species, and deplete pools of the natural intracellular polyamines essential for unlimited proliferation of tumor cells. Furthermore, the potential utility of Nano11047 as a delivery molecule for therapeutic nucleic acids, such as microRNA, is probable based on recent success using the closely related prodrug DSS-BENS [28]. As the antitumor targets of Nano11047 are multiple, the availability of this novel drug form will potentially benefit and encourage future clinical studies.

## Materials and methods

### Synthesis and characterization of Nano11047

Nano11047 was synthesized using protocol similar to our previously described method for BENSpm-based carriers [28, 29]. All reactions were performed in anhydrous conditions under argon atmosphere. First, free base of PG-11047 was obtained by refluxing PG-11047·4HCl (194 mg, 0.48 mmol) with K_2_CO_3_ (495 mg) in 15 mL acetonitrile for 18 h. The suspension was cooled to room temperature, filtered, and the solvent was evaporated under vacuum to afford PG-11047 as a clear colorless liquid (118 mg; 0.46 mmol, 95% yield). Bis(2-hydroxyethyl) disulfide (BHED) was purified on silica gel and 70 mg (0.46 mmol) were dissolved in 0.59 mL dichloromethane (DCM) and 0.12 mL tetrahydrofurane (THF). 1,1’-carbonyldiimidazole (CDI) (156 mg; 0.93 mmol) was suspended in 1.3 mL DCM and the BHED solution was added at 0°C. The reaction mixture turned clear and was kept at 0°C under stirring for 1 h. The progress of the reaction was monitored by thin-layer chromatography (ethylacetate, R_f_ 0.46, UV+ iodine). The reaction mixture was then brought to room temperature and a solution of PG-11047 (118 mg; 0.46 mmol) in DCM (0.63 mL) was added. The reaction mixture became cloudy but clarified when the temperature was increased to 40°C. The reaction was then left to proceed at 40°C for 18 hours. The reaction product was precipitated in diethyl ether, thoroughly washed with cold diethyl ether, dried under vacuum, and dissolved in cold 0.1 mM HCl to convert Nano11047 to its hydrochloride salt. The pH of the aqueous solution was adjusted to 6, and Nano11047 was purified by extensive dialysis (MWCO 1 kDa) against 0.1 mM HCl and water before freeze-drying. The temperature of the dialysis medium was kept at approximately 10°C. The final product was obtained as a white solid at 53% yield (114 mg). ^1^H NMR spectra of Nano11047 in D_2_O were obtained on a Bruker AVANCE-III HD 500 MHz NMR spectrometer and chemical shifts (δ) were expressed in ppm. The molecular weight of Nano11047 was determined by size exclusion chromatography equipped with a TOSOH Bioscience G3000PWxl-CP column eluted in sodium acetate buffer (pH 5). Refractive index (Optilab^®^ T-rEX™) and light scattering (miniDWAN^TM^ TREOS) detectors (Wyatt Technology, Santa Barbara, CA) were used for the detection and Astra 6 software was used to calculate the molecular weight and polydispersity index (PDI). A refractive index increment (dn/dc) of 0.17 mL/g was used for the molecular weight analysis [29].

### Nano11047 degradation kinetics

Degradation kinetics of Nano11047 were determined as previously reported [29]. Briefly, degradation of Nano11047 was monitored by ^1^H-NMR recorded on a 600 MHz Bruker NMR spectrometer and the data were processed by TopSpin 3.5pl6. Nano11047 (9.2 mg) was dissolved in 0.1 M phosphate-buffered D_2_O/acetone-*d6* (3/2, v/v, 0.9 mL, pH 7.4) and purged with argon for 10 min. Dithiothreitol (DTT, 15 mg) was added immediately prior to commencing NMR acquisition at 25°C. The relative decrease in integral intensity of the PG-11047 methylene protons next to the carbamate bond in the Nano11047 (*δ* 3.40–3.70 ppm) was used to determine the degradation. The D_2_O solvent peak observed at 4.80 ppm served as internal standard.

### Cell lines, polyamine analogues, and culture conditions

The human non-small cell lung carcinoma cell lines NCI-H157 (ATCC CRL-5802) and A549 (ATCC CCL-185)(ATCC, Manassas, VA) were maintained in RPMI 1640 medium containing 9% bovine calf serum, penicillin, and streptomycin at 37°C, 5% CO_2_. For all experiments, cells were seeded and allowed to attach overnight. Medium was then aspirated and replaced with that containing polyamine analogue at the indicated concentrations; cells were incubated for the times indicated at 37°C, 5% CO_2_. The polyamine analogue PG-11047 was synthesized by Progen Pharmaceuticals (Queensland, Australia), and *N*^1^,*N*^11^-bis(ethyl)norspermine (BENSpm) was synthesized as previously reported [43]. Stock solutions (10 mM) were prepared in sterile, double-distilled water.

### Cell proliferation assays

For 96-hour experiments, A549 or H157 cells were seeded at 3.5×10^5^ or 7×10^5^ cells per 25-cm^2^ flask, respectively, and allowed to attach overnight. Culture medium was replaced with that containing increasing concentration(s) of Nano11047 or PG-11047, in duplicate. Following incubation for 96 hours, cells were collected by trypsinization and counted using a hemacytometer. Viable cells were determined by their ability to exclude trypan blue.

### RNA extraction and gene expression studies

For gene expression studies, total RNA from analogue-treated cells was extracted using TRIzol reagent (Invitrogen, Carlsbad, CA) according to the provided protocol. RNA was quantified by spectrophotometry, and cDNA was synthesized using qScript cDNA SuperMix (Quanta Biosciences, Gaithersburg, MD). SYBR green-mediated, real-time PCR was performed using primer pairs as previously reported for *SSAT* [44], *SMOX* [45], and *GapDH*. The optimum annealing temperature for each primer pair was determined on cDNA using temperature gradients followed by melt curve analyses and visualization on 2% agarose gels with GelStar staining (Lonza, Walkersville, MD) and KODAK Digital Science Image Analysis Software (Rochester, NY). Amplification conditions consisted of a 5-minute denaturation step at 95°C, followed by 40 cycles of denaturation at 95°C for 30 s, annealing at 60°C for 30 s, and extension at 72°C for 30 s. Universal SYBR Green Supermix was purchased from Bio-Rad (Hercules, CA). Thermocycling was performed on Bio-Rad MyiQ and iQ2 real-time PCR detection systems, with data collection facilitated by the iQ5 optical system software. For each of the qPCR experiments, samples were analyzed in triplicate, normalized to the *GapDH* reference gene, and fold change in expression relative to untreated cDNA was determined using the 2^-ΔΔCt^ algorithm. Custom primers for qPCR were synthesized by Integrated DNA Technologies (Coralville, IA).

### Western blots of polyamine catabolic enzymes

Total protein was extracted from treated cells and quantified using the Bio-Rad DC assay with absorbance measured at 750 nm; values were converted to protein concentration using interpolation on a bovine serum albumin standard curve. Protein samples (30 μg per lane) were separated on pre-cast 4–12% Bis-Tris BOLT gels with 1 × MOPS running buffer (Invitrogen) and transferred onto Immun-Blot PVDF membrane (Bio-Rad). Blots were blocked for 1 hour at room temperature in Odyssey blocking buffer (LI-COR, Lincoln, NE), and proteins of interest were visualized using antibodies specific to SSAT [46], SMOX [45], and ß-actin (Santa Cruz Biotechnology, Dallas, TX). Primary antibodies were diluted 1:1000 in Odyssey blocking buffer containing 0.1% Tween-20, applied to blocked membranes, and incubated at 4°C overnight with rocking. Following washes, blots were incubated for 1 hour at room temperature with species-specific, fluorophore-conjugated secondary antibodies to allow visualization and quantification of immunoreactive proteins using the Odyssey infrared detection system and software (LI-COR).

### Analyses of polyamine catabolic enzyme activity and intracellular polyamine concentrations

Concentrations of intracellular polyamines were determined by pre-column dansylation of cell lysates followed by reverse-phase, high-pressure liquid chromatography, as previously described [47]. 1,7-diaminoheptane was used as an internal control, and individual polyamine concentrations are reported as nmol/mg protein. Measurements of SSAT and ODC enzyme activities were performed according to previously reported methods [32, 48]. SMOX activity was measured using a luminol-based method for the detection of hydrogen peroxide, as previously described [49]. For quantification of the above-mentioned enzyme activity assays, total cellular protein was measured in each sample using the method of Bradford [50].

## Supporting information

S1 Appendix Supporting data files. (XLSX)

S1 Fig NMR signature of Nano11047. (PDF)

## Abbreviations

GSH: glutathione
SSAT: spermidine/spermine *N*^1^-acetyltransferase
SMOX: spermine oxidase
H_2_O_2_: hydrogen peroxide
3-AP: 3-aminopropanal
3-AAP: 3-acetaminopropanal
PG-11047: (*N*^1^,*N*^12^)-bis(ethyl)-*cis*-6,7-dehydrospermine tetrahydrochloride
BESpm: (*N*^1^,*N*^12^)-bis(ethyl)spermine
NSCLC: non-small cell lung cancer
BENSpm: (*N*^1^,*N*^11^)-bis(ethyl)norspermine
ODC: ornithine decarboxylase
OAZ: ODC antizyme
