# Correction: Biochemical evaluation of the anticancer potential of the polyamine-based nanocarrier Nano11047

**DOI:** 10.1371/journal.pone.0178102

**Published:** 2017-05-16

**Authors:** Tracy Murray-Stewart, Elena Ferrari, Ying Xie, Fei Yu, Laurence J. Marton, David Oupicky, Robert A. Casero

In Fig 2, the molecules PG11047 and Nano11047 are mistakenly drawn as trans isomers when they should be cis isomers. Please view the corrected Fig 2 here.

**Fig 2:**
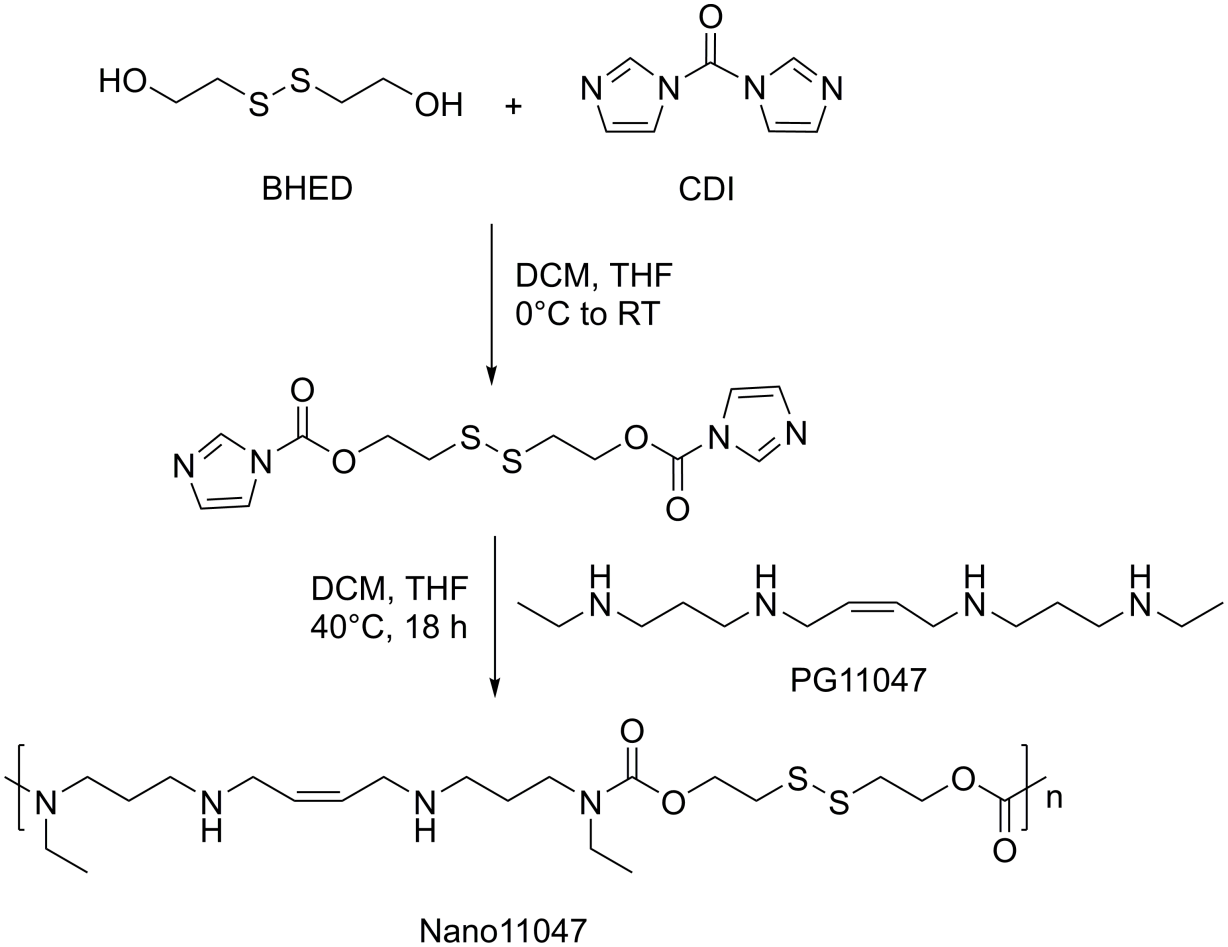
Synthesis of Nano11047.
