# Supplementary figures and images for: Biochemical evaluation of the anticancer potential of the polyamine-based nanocarrier Nano11047
Source: PLoS One. 2017 Apr 19;12(4):e0175917. doi: 10.1371/journal.pone.0175917 (PMC5396973; doi:10.1371/journal.pone.0175917)

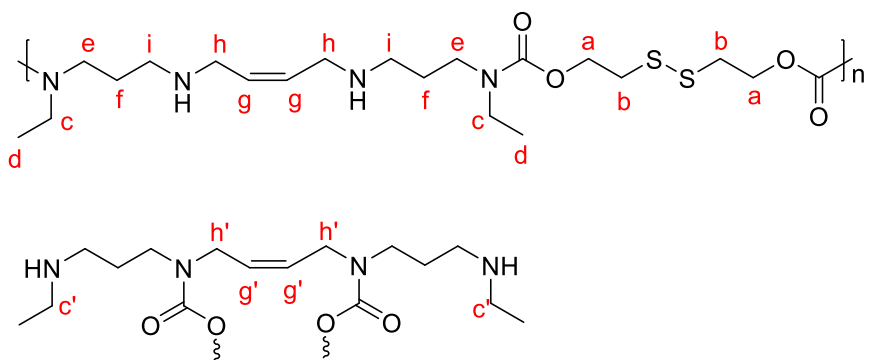

5.55-6.05 (2H, **g and g'**), 4.41 (4H, **a**), 3.75-4.20 (4H, **h and h'**), 2.90-3.55 (16H, **b, c, c', e, e' i, i'**), 1.99 (4H, **f**), 1.05-1.38 (6H, **d**)

**SI Fig 1. NMR signature for Nano11047**

Supplement: S1 Fig — (PDF) [file pone.0175917.s002.pdf]
